# Visual Hallucinations from Zolpidem Use for the Treatment of Hospital Insomnia in a Septuagenarian

**DOI:** 10.7759/cureus.3848

**Published:** 2019-01-08

**Authors:** Raza Mian

**Affiliations:** 1 Internal Medicine, University of California San Francisco, Fresno, USA

**Keywords:** zolpidem, ambien, hallucination, insomnia

## Abstract

Insomnia and parasomnias are common patient complaints during hospital stay. New environment, the severity of underlying disease, level of care, medications, and infections are all known factors that contribute toward insomnia. Zolpidem is a common sleep aid used for this purpose. We report a case of Zolpidem-induced visual hallucination in a septuagenarian in the inpatient setting.

## Introduction

Insomnia is a common complaint in the inpatient setting. Increasing age, disease severity, underlying psychiatric condition, pain, location, and medications contribute to insomnia. Therapies include non-pharmacological and pharmacological interventions. Sleep aids make the cornerstone of pharmacological interventions. The use of sleep aids in the inpatient setting is also common. These include sedating antihistamines, melatonin, antidepressants, and antipsychotics. Zolpidem is a commonly used sleep aid. We report a case of Zolpidem-induced visual hallucination in a patient in his seventies.

## Case presentation

A septuagenarian patient presented to the hospital with a complaint of worsening back pain. He had a pertinent previous medical history of recently diagnosed multiple myeloma. He reported sudden-onset, non-traumatic, spontaneous, mid-to-low, non-radiating, midline back pain that started two weeks ago. He denied association with bladder or bowel dysfunction, lower-extremity weakness, or paresthesia. His primary care provider had prescribed him non-steroidal anti-inflammatory agents without significant improvement. On presentation, he remained afebrile with normal vital signs. He had no saddle analgesia and a normal rectal tone. Post-void urinary bladder ultrasound revealed an empty bladder. He had a normal and symmetric sensation to light touch, vibration, and temperature. Patellar reflexes were normal and he had a normal Babinski's test. Hematological lab tests revealed microcytic anemia. Imaging studies revealed a compression fracture of the ninth and tenth thoracic vertebrae. Neurosurgery evaluated the patient recommending against surgical fixation. A spinal brace was provided to assist with comfort. Radiation oncology evaluated the patient and a decision was made to pursue radiation therapy.

The patient complained of insomnia during the hospital stay and was prescribed Zolpidem of 5 mg. At six o'clock, the next morning, the patient was noticed by nursing staff to be delirious reporting seeing graphic looking cats on the wall. Conservative measures and reorientation led to the subsequent resolution of the symptoms over a period of 10 minutes. The patient had complete recollection of the event. During the subsequent hospital stay, no further doses of Zolpidem were administered. No further episodes of visual hallucination occurred. 

## Discussion

Insomnia is a common patient complaint in the inpatient setting [1]. Various risk factors are known in this setting, which can contribute to insomnia, including disease severity, poor quality or amount of sleep, new environment, and disturbed wake-sleep cycle, lack of exercise, medications, and infections [2]. Hospital-acquired delirium shares some of the same risk factors [3]. Treatment is sought by employing non-pharmacological and pharmacological tools. These include improved daytime lighting including sunlight exposure, ambulation, reduced nighttime disturbance, and continued treatment of the underlying disease process [4]. However, many times, non-pharmacological aids are supplemented by pharmacological agents. Commonly employed agents include melatonin, sedative-hypnotics, antidepressants, and antipsychotic agents [5]. Zolpidem is a commonly used sedative-hypnotic.

The common side effects of Zolpidem include a headache, drowsiness, dizziness, lethargy, depression, and constipation. Severe side effects of Zolpidem include suicidal ideation, aggressive behavior, impaired mental alertness the following day, hallucinations, amnesia, anaphylaxis, angioedema, and withdrawal [6-9].

This case report discussed visual hallucination in a patient with risk factors associated with disturbed inpatient sleep and hospital delirium. Zolpidem was used as a sleep aid. Subsequently, hallucinations and delirium were noticed. Brodeur et al. reported a case of an octagenarian patient with a similar hallucination side effect; however, the symptoms lasted longer [10]. The impact on patient health during these episodes can be varied. There have been reports of somnambulism as well as homicide [11]. Advanced, female gender, concomitant use of selective serotonin reuptake inhibitors (SSRI), and Zolpidem dosage >10 mg have been reported to be associated with hallucinogenic side effects of Zolpidem; however, it can be seen in the younger population as well [10-13].

Although relatively safe, it is important to identify and recognize the potential side effects of this drug. Caution is advised in the at-risk population.

## Conclusions

Insomnia is a common inpatient complaint. Symptomatic management with the use of sleep aids is common, and Zolpidem is a commonly used agent. Visual hallucinations can occur after Zolpidem administration. At-risk patients should avoid Zolpidem as a sleep agent.
